# Spatially Resolved Profiling of Compartmentalized Muscle and Brain Inflammation

**DOI:** 10.1002/eji.70119

**Published:** 2025-12-22

**Authors:** Thorge Dobbertin, Lucas Schirmer

**Affiliations:** ^1^ Division of Neuroimmunology, Department of Neurology, Medical Faculty Mannheim Heidelberg University Mannheim Germany; ^2^ Faculty of Biosciences Heidelberg University Heidelberg Germany; ^3^ Interdisciplinary Center for Neurosciences (IZN) Heidelberg University Heidelberg Germany; ^4^ Mannheim Center for Translational Neuroscience (MCTN) and Institute for Innate Immunoscience (MI3), Medical Faculty Mannheim Heidelberg University Mannheim Germany

**Keywords:** inflammation, immunopathology, neuroimmunology, proteomics, transcriptomics

## Abstract

Spatial omics technologies enable high‐resolution mapping of transcriptomic, proteomic, and metabolic profiles within intact tissues, revealing how immune, stromal, and parenchymal cells interact in situ during inflammation. Chronic inflammation in skeletal muscle and the central nervous system is spatially organized within defined niches that shape disease progression and therapeutic response. In skeletal muscle, spatial analyses have uncovered fiber‐type‐specific vulnerability, regenerative trajectories, and immune–stromal crosstalk in disorders such as Duchenne muscular dystrophy and inclusion body myositis. In the central nervous system, these approaches have revealed compartmentalized neuroinflammation in multiple sclerosis, innate immune activation in amyotrophic lateral sclerosis, and immune evasion in glioma. Integration with single‐cell gene expression enables inference of cell–cell communication networks and identification of spatial gradients of immune activation and tissue remodeling. Despite major advances, clinical translation remains limited by small cohorts, methodological variability, and insufficient functional validation. As spatial profiling becomes more accessible, standardized, and scalable, it promises to stratify inflammatory disease states, identify tissue‐resident immune programs, and guide mechanism‐based therapies. Hence, spatial omics provide an unprecedented opportunity to resolve the cellular architecture of inflammation, revealing not only where immune activity occurs, but how it is orchestrated within complex tissue microenvironments.

## Graphical abstract

This review summarizes emerging spatially resolved multi‐omics approaches revealing organized cell–cell interactions in skeletal muscle and brain inflammation. These tools uncover radiating molecular programs and niche‐specific immunopathology that shape cellular reactivity and vulnerability.

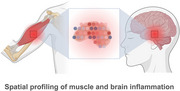

**Abbreviations**
5′RACE: 5′ rapid amplification of cDNA ends
AD: Alzheimer's disease
ALS: amyotrophic lateral sclerosis
BCR: B cell receptor
CCC: cell–cell communication
CD: cluster of differentiation
CNS: central nervous system
DMD: Duchenne muscular dystrophy
DSP: digital spatial profiling
EAE: experimental autoimmune encephalomyelitis
EC: endothelial cell
FAP: fibro‐adipogenic progenitor
FISH: fluorescence in situ hybridization
H&E: hematoxylin and eosin
HSP: heat shock protein
IF: immunofluorescence
IHC: immunohistochemistry
IIM: idiopathic immune‐mediated myopathy
IMC: imaging mass cytometry
ISH: in situ hybridization
ISS: in situ sequencing
LCM: laser capture microdissection
MHC: major histocompatibility complex
MIBI: multiplexed ion beam imaging
MS: multiple sclerosis
MSI: mass spectrometry imaging
MYH: myosin heavy chain
NGS: next‐generation sequencing
NMJ: neuromuscular junction
RNA‐seq: RNA sequencing
scRNA‐seq: single cell RNA‐sequencing
snRNA‐seq: single‐nucleus RNA‐sequencing
TCR: T cell receptor

## 1 Introduction

Cells in the human body are not randomly distributed but function within specialized microenvironments or niches. In chronic inflammatory diseases such as idiopathic immune‐mediated myopathies (IIMs) and multiple sclerosis (MS), inflammation is not diffuse but compartmentalized, localizing to distinct tissue regions characterized by specific cellular compositions and signaling dynamics [1, 2, 3]. These immune niches are shaped by both intrinsic genomic regulation and context‐specific intracellular signaling pathways. Over the past decade, advances in single‐cell technologies have enabled detailed profiling of cellular transcriptomes, allowing researchers to define cell types and states with high precision. However, most bulk and single‐cell methods rely on tissue dissociation, resulting in the loss of critical spatial information.

Spatial omics technologies have emerged to fill this gap, offering the ability to map molecular profiles directly within the intact tissue architecture [4, 5, 6, 7]. This enables high‐resolution analysis of cells within their native microenvironment and the elucidation of local cellular interactions. While spatial omics have primarily focused on the transcriptome, there is growing momentum toward spatially resolving additional molecular layers, including the (epi‐)genome, proteome, lipidome, glycome, and metabolome. These multidimensional spatial readouts allow researchers to:

- define and classify cellular neighborhoods, and
- identify interaction networks across different cell types and molecular modalities [8, 9, 10, 11].

Despite recent improvements in spatial profiling depth and resolution [7, 12, 13, 14, 15], these methods often demand significant resources and time, particularly when compared with nonspatial bulk or single‐cell omics. Nevertheless, the added spatial dimension is critical for understanding not just which cells are present, but how they interact—physically, functionally, and molecularly—within organized tissue compartments. Importantly, intercellular networks often span across spatial domains and molecular layers, making integrative multimodal approaches essential. As a result, there is increasing interest in combining spatial and nonspatial datasets to maximize biological insight [16, 17, 18, 19].

In this review, we highlight recent methodological and conceptual advances in spatially resolved profiling, with a focus on their application to muscle and brain tissues. These highly structured and functionally complex organs are particularly well suited to spatial approaches, especially in the study of inflammation, degeneration, and regeneration [20, 21, 22, 23, 24, 25]. Spatial analysis has revealed how dynamic cellular states evolve in response to disease or injury, and how immune cells interact with resident cell types such as myofibers, neurons, glia, and stromal cells. Such interactions are often central drivers of disease‐specific pathology [1, 3, 26, 27, 28, 29], underlining the need to move beyond mere immune cell enumeration toward a more holistic understanding of immune‐tissue communication. We present and discuss recent studies that leveraged spatial profiling to gain systems‐level insights into organized tissue compartments in the context of chronic inflammation. These examples underscore the power of spatial approaches in elucidating niche‐specific mechanisms of immune crosstalk and tissue remodeling in both health and disease.

## 2 Advancing Omics through Spatial Methodologies

Histological slides remain a cornerstone of clinical diagnostics and prognostics, serving as the gold standard in routine medical practice. With the rise of omics technologies, there is a growing effort to enhance conventional tools by integrating molecular data, thereby generating fully profiled, spatially annotated tissue maps. Achieving this goal, however, still faces key challenges, including (a) limited spatial resolution, (b) constrained multiplexing and throughput, and (c) the complexity of multimodal data integration.

### 2.1 Spatial Transcriptomics

A major breakthrough came in 2016, when next‐generation sequencing (NGS) was first combined with spatial barcoding, enabling transcriptome‐wide profiling within tissue sections [4]. Initial techniques, such as barcoded solid‐phase RNA capture, were limited to ∼100 µm resolution. However, rapid advances have led to high‐resolution platforms like HDST, Slide‐seqV2, and Visium‐HD, now achieving resolutions of 2–20 µm [5, 6, 7, 12]. More recently, methods such as Stereo‐seq, Seq‐Scope, and PIXEL‐seq have pushed the limits further, reaching submicron resolution (∼0.2–1.22 µm) [13, 14, 30]. Importantly, these nominal resolutions are often based on the physical size of barcoded capture features, which do not necessarily reflect the true biological resolution due to molecular diffusion and capture variability [14, 15].  An alternative strategy involves in situ hybridization (ISH), which enables direct tagging of RNA molecules using fluorophore‐labeled probes for visualization, as seen in commercial systems like Xenium (formerly CartaNA), MERSCOPE, and CosMx SMI, as well as academic platforms including MERFISH [31], EEL fluorescent in situ hybridization (FISH) [32], and STARmap PLUS [33]. While many of these platforms, like STARmap and Xenium, also employ in situ sequencing (ISS) chemistries, they differ in their resolution, sensitivity, and compatibility with various tissue types [34]. ISH can theoretically reach single‐molecule resolution but is limited by optical constraints, probe design, and the need for preselected gene panels, often focusing on cell type‐specific markers and, hence, not covering the whole transcriptome. Computational approaches such as PERSIST [35] aim to optimize gene panel design, yet trade‐offs between resolution, transcriptome coverage, and discovery potential remain.

### 2.2 Mapping Intercellular Communication

In parallel to improved spatial resolution and multiplexing depth, spatial transcriptomic data are increasingly leveraged to dissect cell–cell communication (CCC) within complex tissue environments. Cellular interactions can be inferred from the gene expression of known ligand–receptor pairs and mapped to specific tissue niches, thereby elucidating how cells communicate within their microenvironment. Although CCC analysis involves physical interactions and metabolites, these elements are partially reflected in molecular signaling. With the increasing availability of data, particularly transcriptome and protein–protein interactions cataloged in databases, various computational methods have been developed to predict interactions within datasets [11]. Several computational frameworks, like LIANA, which aggregates multiple CCC tools [36], CellChat, focusing on pathway‐level activity [37], and NicheNet, linking ligands to downstream targets [38], offer robust solutions to predict CCC events. Signaling pathways not only facilitate the definition of intra‐ and intercellular communication within a tissue but also help explain how cellular niches respond to environmental cues, tissue injury, and inflammation. Initially, tools were designed for bulk data from homogenized tissue or single‐cell data from dissociated tissue and then applied to spatial data [8]. More are continuously being developed specifically for spatial data [9, 10], incorporating information on cell proximity, gradients, and anatomical boundaries. Although spatial profiling enables colocalization of ligand and receptor pairs, follow‐up functional studies are generally needed to validate biological inference.

### 2.3 Spatial Proteomics and Metabolomics

Despite the widespread adoption of transcriptomic profiling, its capability to infer protein levels remains debated and is not sufficient to fully describe individual cell states [39]. For a more complete understanding of cellular processes and relationships, the use and implementation of other modalities beyond transcriptomics is highly beneficial, such as cell‐type‐specific and spatially resolved proteomics, lipidomics, and metabolomics. Spatial proteomics offers a more immediate, functional readout of cell state. Traditional methods such as immunofluorescence (IF) and immunohistochemistry (IHC) are well established but limited in throughput and multiplexing. Newer techniques, including sequential immunofluorescence like COMET [40], CODEX/Phenocycler [41], imaging mass cytometry (IMC) [42], and multiplexed ion beam imaging (MIBI) [43, 44], now enable the simultaneous visualization of dozens to over 100 proteins in a single tissue section. A promising frontier lies in dual transcriptomic–proteomic profiling from the same tissue section, as shown via the commercial GeoMx DSP [45] platform, enabling direct comparison of spatially resolved RNA and protein expression. While antibody‐based approaches depend on predefined panels that constrain discovery, mass spectrometry‐based methods such as laser capture microdissection (LCM) followed by LC‐tandem mass spectrometry offer label‐free quantification of proteins, endogenous metabolites, and lipids despite generally lower spatial coverage [46]. At the cost of molecular depth, Mass spectrometry imaging (MSI) achieves generally higher spatial resolution in situ, as shown in both neurological and neuromuscular disorders [47, 48].

### 2.4 Computational Integration and Future Directions

**FIGURE 1:**
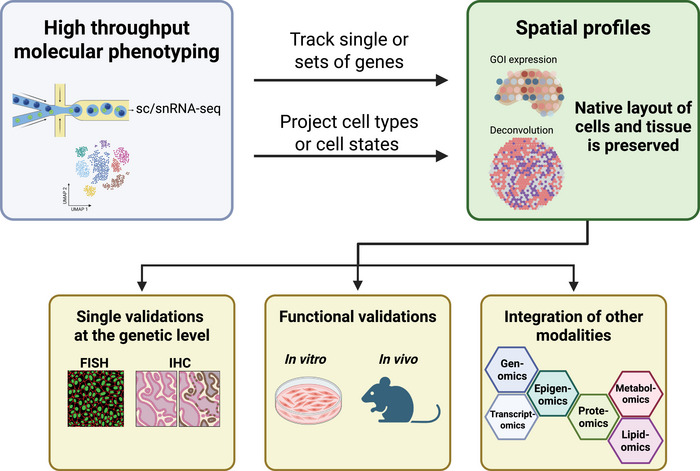
Conceptual framework for spatially informed tissue profiling. High‐throughput molecular phenotyping via single‐cell or single‐nucleus RNA sequencing enables the characterization of expression in each cell. Cell types or states can be clustered following dimensional reduction, most commonly UMAP. Individual genes or small subsets can be localized in spatial transcriptomic datasets. At the same time, transcriptional identities (like cell types) can be projected onto spatial maps, integrating both modalities. From there, several downstream applications may be used. Including [1] Single gene validations using orthogonal imaging‐based methods like FISH or IHC [2] Functionally validate spatial findings in culture models in vitro or in animal models in vivo [3] Expand the analysis to incorporate other modalities (omics) that in turn may be integrated. Created with Biorender.com.

Complementing these experimental advances, computational frameworks have evolved to analyze and integrate spatial omics data. These datasets are not only large but also multimodal, containing image data, coordinate matrices, and diverse molecular profiles. This demands scalable, spatially aware analytical tools for robust downstream analysis. A fundamental step is cell type deconvolution, where spot‐level transcriptomes (typically from spatial transcriptomics platforms) are linked to single‐cell or single‐nucleus RNA‐seq reference datasets [49]. Tools like Cell2location [17], Tangram [16], and Seurat [18] have been used to map neuronal subtypes across cortical layers or immune infiltration patterns in muscle tissues. This may be followed by gene/protein validations or additional modalities to get a robust, complete spatial tissue profile (Figure 1). Of note, for higher‐resolution spatial methods, subcellular segmentation is needed to assign molecules to individual cells [19, 50]. In tissues or experimental settings where segmentation is impractical, alternative approaches such as pseudo‐cell localization can be employed to map expression patterns [51]. There is also increasing demand for integrative analysis across different modalities and for standardized, reproducible pipelines across experiments and datasets. Frameworks like SpatialData [52] and PysoDb [53] address this gap by offering open, extensible infrastructures for the storage, annotation, and analysis of spatially resolved multi‐omics data. These technologies represent only a snapshot of a rapidly evolving field. As spatial omics methods continue to improve in resolution and scalability, researchers must carefully match experimental design—including tissue type, resolution needs, and throughput—to the biological questions. In this review, we highlight recent applications of spatial molecular profiling in muscle and brain, two structurally complex and functionally dynamic organs. We explore how these tools have advanced our understanding of chronic inflammation at the systems level.

**Spatially resolved adaptive immune cell profiling**

Clonal expansion and tissue infiltration of lymphocytes, such as T and B cells, are hallmark features of antigen‐driven, adaptive immune responses. The adaptive immune system is characterized by its specificity for antigenic determinants, typically short peptides presented via major histocompatibility complex (MHC) molecules, and its ability to establish long‐lasting immunological memory.

To recognize the vast diversity of potential antigens, T and B cells express highly variable receptors (TCRs and BCRs), generated through somatic recombination of variable (V), diversity (D), and joining (J) gene segments. Each lymphocyte clone expresses a unique receptor defined primarily by the hypervariable complementarity‐determining region 3 (CDR3), which forms at the V(D)J junction and dictates antigen specificity. As a result, unlike other immune cell types that can be classified by shared marker gene expression, T and B cells require V(D)J sequencing to resolve their clonal identity and diversity [54]. Importantly, the composition of a receptor repertoire of an individual can provide insights into disease states; for example, TCR and BCR sequence data alone have been used to predict viral infections with high accuracy [55]. Integrating receptor repertoire profiling with spatial information offers powerful insights into the tissue organization of adaptive immune responses. Spatial mapping of TCR/BCR sequences enables the identification of immune niches and the analysis of lymphocyte behavior in situ, such as clonal expansion near antigen sources, the spatial trajectory of immune cell infiltration, and B cell maturation within germinal centers. However, most conventional transcriptomic protocols rely on 3′‐end sequencing strategies optimized for poly‐A tail capture. These approaches fail to recover the critical 5′ variable regions of TCR and BCR transcripts, where the clonotype‐defining sequences reside.

Two major technical challenges limit the recovery of V(D)J information from spatial data: first, receptor transcripts are typically expressed at low levels relative to the whole transcriptome, reducing their detectability. Second, the V(D)J region lies upstream of a long 3′ constant region, often making it inaccessible in standard 3′ capture methods. Consequently, many spatial transcriptomics platforms lose essential clonotype information, diminishing their utility for adaptive immune profiling. Deep sequencing can partially mitigate low transcript abundance but is resource‐intensive and may increase background noise, complicating downstream error correction and analysis. To address these issues, most workflows incorporate targeted V(D)J enrichment strategies. These include PCR amplification of less variable J or C regions, 5′‐Rapid Amplification of cDNA Ends (5′RACE), and hybrid capture approaches. Such enrichment enables more accurate detection of clonally expanded lymphocytes, tracking of differentiation trajectories, and functional characterization of immune responses [56]. Capture‐based enrichment has also been successfully combined with long‐read sequencing (e.g., Oxford Nanopore) to obtain full‐length TCR/BCR sequences [54]. More recently, enrichment protocols have been adapted for spatial applications, allowing for localized mapping of TCR [57, 58] and combined TCR/BCR repertoires [59].

Despite these advances, several critical challenges persist. Labeling strategies must be further refined to improve the capture efficiency of low‐abundance receptor transcripts without introducing technical bias. Spatial resolution must be sufficient to resolve closely adjacent lymphocytes sized ∼7 µm [60], particularly in dense inflammatory infiltrates. Moreover, integrated computational frameworks capable of linking spatial position, clonotype identity, and transcriptional state into coherent models of immune organization are still in development [61]. Meeting these challenges will be essential for fully leveraging spatial omics technologies to decode adaptive immune responses in the tissue context.

## 3 Spatial Mapping of Muscle Inflammation

**FIGURE 2:**
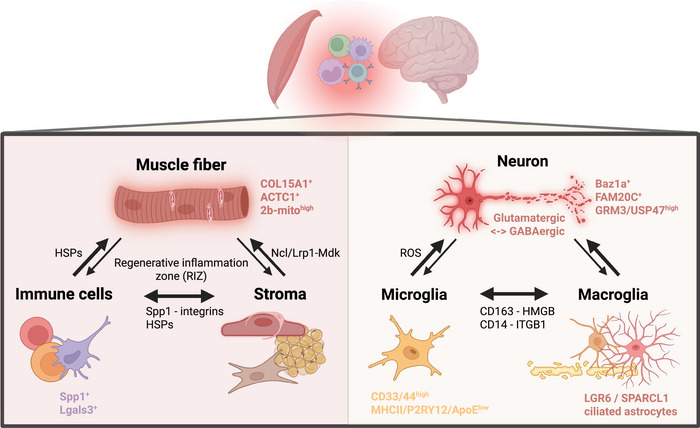
Spatial and molecular communication in (inflamed) brain and muscle tissue. Muscle fibers interact with both immune cells (like lymphocytes, macrophages, and neutrophils) and stromal cells (like fibroblasts, endothelial cells, pericytes, and adipocytes). Similarly, neurons engage with both proximal microglia and macroglia (like oligodendrocytes, astrocytes, and ependymal cells). These interactions present a dynamic network where infiltrating immune cells impair tissue function and repair, while tissue damage in turn shapes immune activation and remodeling. HSPs: heat shock proteins. Created with Biorender.com.

Skeletal muscle is a highly specialized and structurally organized tissue composed of diverse cell types, including innervated myofibers, fibroblasts (Fb), endothelial cells (EC), pericytes, resident and infiltrating immune cells, and muscle stem/progenitor cells (Figure 2). The myofibers, consisting of multinucleated, contractile cells expressing myosin heavy chain (MYH), serve as the primary functional units of muscle tissue. These fibers exhibit functional heterogeneity, with distinct MYH isoforms defining different fiber types: “slow” oxidative Type 1 fibers express MYH7, while the “fast” but more fatigue‐prone Type 2 fibers express isoforms such as MYH2, MYH4, and MYH1. Myofibers also differ in metabolic properties, with Types 1 and 2A being oxidative and Types 2B and 2X primarily glycolytic. The spatial organization of skeletal muscle is equally critical to its function. Myofibers are ensheathed by the endomysium, grouped into fascicles surrounded by the perimysium, and embedded within a hierarchical extracellular matrix. Additional structures, ranging from the sarcolemma and internal myofibrils to the vascular and neuromuscular networks, contribute to tissue integrity, metabolic support, and regenerative capacity.

Recent advances in spatial omics technologies have provided unprecedented insight into the maintenance, remodeling, and pathological disruption of these tissue architectures (Table 1). For example, studies have revealed the expansion and altered signaling of fibro‐adipogenic progenitor cells (FAPs) during muscle regeneration. In myotoxin‐induced injury models, researchers identified novel transitional myogenic progenitor cell populations (“committed”, “fusing” myogenic cells) bridging classical Pax7 quiescence to Acta1 maturation [20]. They further integrated sc/snRNA‐seq data with Visium spatial transcriptomics to map spatiotemporal gradients of myogenesis in the days past injury (dpi) at the injury zone. This included coordinated stromal‐immune remodeling marked by an increase of Pdgfra‐expressing FAPs and a decrease of patrolling M2‐like monocytes 5–7 dpi. CCC analysis using CellChat further demonstrated increased spatial co‐expression of *Mdk* (FAPs) and its receptors *Ncl/Lrp1* (myogenic cells) during regenerative responses. Similarly, in Duchenne muscular dystrophy (DMD) mouse models, integrated Visium spatial and single‐cell transcriptomics revealed FAP expansion and CCC networks emanating from dystrophic regions, with distinct inflammatory (*Ccl2*, *Ccl7*, and *Spp1*) and regenerative‐like enriched collagen expressing FAPs clusters [21]. Taken together, the integration of spatial data revealed emerging myocyte and FAP cell states during muscle regeneration and inflammatory signals radiating from injury zones to healthy tissue, promoting pervasive fibrosis.

**TABLE 1:** Overview of spatially informed studies in skeletal muscle and the central nervous system.

Skeletal muscle tissue							
Title of publication	First author	Year of publication	Spatially resolved methods	Species	Context/disease	Tissue type	Main results
Large‐scale integration of single‐cell transcriptomic data captures transitional progenitor states in mouse skeletal muscle regeneration	David McKellar	2021	Spatial transcriptomics (**Visium** 2–7 dpi) integrated with scRNA‐seq & snRNA‐seq (0–21 dpi)	Mouse (4–7 months)	Induced Injury (notexin & cardiotoxin) Regenerative myogenesis	Tibialis muscle	Identified novel, rare, short‐lived progenitor states and transitional cell subtypes in regenerating muscle
Spatial proteomics reveals heterogeneity in neural markers underpinning high‐fat diet‐induced myopathy in male mice	Lydia Hardowar	2023 (preprint)	Spatial proteomics (**GeoMx**), IF	Mouse	High‐fat diet–induced metabolic myopathy (12 weeks of feeding)	Tibialis muscle	Structural damage correlates with inflammatory infiltration and maladaptive muscle remodeling Expression of neuronal markers, indicating denervation, in desmin fibers
Regenerating human skeletal muscle forms an emerging niche in vivo to support PAX7 cells	Michael R. Hicks	2023	Spatial transcriptomics (**GeoMx** 60–120 dpi) paired with snRNA‐seq (8 dpi), IF, histology	Mouse chimeric model (Human cells engrafted into mouse host)	Muscle regeneration in DMD + cardiotoxin re‐injury (60 dp first injury)	Human SMPCs engrafted into mouse tibialis muscle	ACTC1‐expressing myofiber interacts with PAX7 SMPCs to enable muscle regeneration in niches ACTC1 depletion also reduces number of SMPCs
Single‐cell and spatial transcriptomics identify a macrophage population associated with skeletal muscle fibrosis	Gerald Coulis	2023	Spatial transcriptomics (**Visium**) combined with scRNA‐seq, IF	Mouse (4–52 weeks) Human	DMD VCP DUX4 Inflammatory (IBM, ASS, IMNM) and dystrophic (DMD, LGMD) myopathies	Quadriceps	Macrophage clusters not matching classical M1/M2 states Profibrotic macrophage cluster with characterizing elevated *Gal‐3* (*GAL‐3*) expression also found in (muscle disease) human cell population
Spatial metabolomics reveals skeletal myofiber subtypes	Lanfang Luo	2023	Spatial Metabolomics (**MALDI‐MSI**) paired with LC‐MS, IF	Mouse (4–6 months)	Physiological muscle (fiber‐type profiling)	Gastrocnemius and soleus	Fiber metabolic phenotypes Novel myofiber subtype with hybrid fatty acid oxidative and glycolytic metabolism
A cellular and molecular spatial atlas of dystrophic muscle	Michael J. Stec	2023	Spatial transcriptomics (**Visium** 1–5 dpi) integrated with scRNA‐seq	Mouse (6 weeks or severely dystrophic)	DMD Induced injury by cardiotoxin	Gastrocnemius/plantaris muscle complex	Propagation of inflammatory and regenerative signals in discrete spatial patches with outspreading CCC Targets for DMD study and therapy (like collagens, *Spp1*)
Spatiotemporal transcriptomic mapping of regenerative inflammation in skeletal muscle reveals a dynamic multilayered tissue architecture	Andreas Patsalos	2024	Spatial transcriptomics (**Visium** 4–8 dpi) combined with scRNA‐seq (1–2 dpi), IF, and histology	Mouse (2 months)	DMD Induced injury by cardiotoxin	Tibialis muscle	Identified spatial organization of monocyte/macrophage and DC subtypes into regenerative inflammation zones (RIZs) that coordinate clearing and myogenesis; glucocorticoid treatment disrupts these zones Dendritic cell and (anti‐inflammatory) macrophage subsets
Spatial multi‐omics in whole skeletal muscle reveals complex tissue architecture	Clara Martínez Mir	2024	3D spatial transcriptomics (**TOMOseq**) integrated with spatial metabolomics, lipidomics (**MALDI‐MSI**), RNA‐FISH validations	Mouse (4–6 months)	Healthy muscle	Tibialis muscle	Proximal‐distal myofiber distribution with distinct metabolic, lipidomic, and gene expression zones Integrated analysis of spatial transcriptome and metabolome across fiber types
Cell type mapping of inflammatory muscle diseases highlights selective myofiber vulnerability in inclusion body myositis	Sven Wischnewski	2024	Spatial transcriptomics (**Visium**) integrated with snRNA‐seq RNA‐FISH validations	Human	IBM IMNM	Quadriceps	Selective type 2A fiber vulnerability in IBM Genomic and protein stress‐associated pathways driving IBM Localization of immune and profibrotic niches
High‐resolution spatial transcriptomic atlas of mouse soleus muscle: Unveiling single cell and subcellular heterogeneity in health and denervation	Jer‐En Hsu	2024 (preprint)	Spatial Transcriptomics (**Seq‐Scope** 3–7 dpi) paired with Histology	Mouse (6 months)	Denervation (surgical nerve removal)	Soleus (with and without sciatic nerve)	Novel hybrid fibers at a transitional state Mapped NMJ nuclei Fiber differences to stress and Denervation‐induced ECM remodeling dynamics
Transcriptomic analysis of skeletal muscle regeneration across mouse lifespan identifies altered stem cell states	Lauren D. Walter	2024	Spatial transcriptomics (**Slide‐Seq** 5 dpi) integrated with scRNA‐seq (0–7 dpi)	Mouse (4–26 months)	Induced injury by notexin	Tibialis muscle	Spatial atlas from 4–26 months old mice Resident and immune cell lineages with differences across age groups Senescent‐like Stem cell subset in older mice impairing regeneration
In situ spatial transcriptomic analysis of human skeletal muscle using the Xenium platform	Nejc Umek	2025	Spatial transcriptomics (**Xenium**) combined with IHC	Human	Aging	Quadriceps	DEGs for fiber types, location (fiber vs perimysium), and age Higher transcript density confirmed in Type 1 fiber, especially in nuclear proximity

Central nervous system							
Title of publication	First author	Year of publication	Spatially resolved methods	Species	Context/disease	Tissue type	Main results
Spatiotemporal dynamics of molecular pathology in amyotrophic lateral sclerosis	Silas Maniatis	2019	Spatial transcriptomics (**ST**), FISH validations	Mouse (SOD1‐G93A) Human	ALS	Spinal cord (lumbar, cervical)	Spatially vulnerable spinal cord niches manifest during disease progression Innate immune response and neuroinflammation are followed by oxidative stress, protein misfolding, and complement cascade Molecular modules (like complement‐, microglia activation, and Interferon signaling) shared between advanced stage ALS mouse model and human tissue
Transcriptome‐scale spatial gene expression in the human dorsolateral prefrontal cortex	Kristen R. Maynard	2021	Spatial transcriptomics (**Visium**) integrated with snRNA‐seq RNA‐FISH validations	Human	Healthy brain coordination	Dorsolateral prefrontal cortex	Cortical layer enriched gene signatures (like autism associated genes enriched in layer V) implicate intracortical information processing as a potential mechanism for genetic risk Full cortical depth and underlying white matter mapped
Dense functional and molecular readout of a circuit hub in sensory cortex	Cameron Condylis	2022	Two‐photon calcium imaging followed by spatial transcriptomics (**multiplexed FISH**) "CRACK" platform	Mouse (6–8 weeks)	Sensory processing during behavior	Primary somatosensory cortex	Inhibitory and excitatory neuronal subclasses with *Baz1a*‐expressing neurons as sensory‐driven circuit organizers Platform to map both functional and transcriptional neuronal cell states in situ
T‐cell dysfunction in the glioblastoma microenvironment is mediated by myeloid cells releasing interleukin‐10	Vidhya M. Ravi	2022	Spatial transcriptomics (**Visium**) integrated with scRNA‐seq	Human	Immunosuppressive T‐cell exhaustion in glioblastoma	Tumor tissue (neuroglia)	Myeloid cells expressing *HMOX1* and releasing immunosuppressive IL‐10 are enriched in mesenchymal like tumor region Exhausted T cells also localize with such mesenchymal tumor compartments, but exhaustion is reversible, blocking IL‐10 or JAK‐STAT signaling downstream
Spatiotemporal molecular dynamics of the developing human thalamus	Chang N. Kim	2023	Spatial transcriptomics (**MERSCOPE**) integrated with scRNA‐seq	Human	Thalamic development	Prenatal thalamus	Differentiation trajectories of thalamic neurons Developmental timepoints in fetal thalamus, like Gluta‐ and GABAergic neurons, during the first trimester Spatial gradients during glial cell maturation
Spatial transcriptomics reveal neuron‐astrocyte synergy in long‐term memory	Wenfei Sun	2023	Spatial transcriptomics (**MERSCOPE**) integrated with scRNA‐seq, RNA‐FISH validations	Mouse (49–56 days)	Long‐term fear memory	Basolateral amygdala	Core memory engram formed by *Penk*⁺/*Tac* ^−^ neurons interacting with adjacent astrocytes, also showing memory‐associated gene signatures Functionally interactive niche with spatial proximity of involved cells necessary for memory consolidation, as verified in loss‐of‐function assays
Cellular architecture of evolving neuroinflammatory lesions and multiple sclerosis pathology	Petra Kukunja	2024	Spatial transcriptomics (**Cartana** and **Xenium**) IHC, IF validations	Mouse (EAE) (9–13 weeks) Human	MS	Spinal cord, select brain regions	Disease‐associated glia cells also identified in lesion‐free regions Radial rim architecture with immune core that is resolved in later lesion stages, while glial shell remains
Spatially resolved gene signatures of white matter lesion progression in multiple sclerosis	Astrid M. Alsema	2024	Spatial transcriptomics (**GeoMx, Visium, and Cartana**) integrated with snRNA‐seq IHC validations	Human	MS	Subcortical lesions	White matter molecular lesion rims even in absence of MRI features Molecular trajectories from white matter into different lesion types with a focus on transitional activated/inactivated lesions
Cell type mapping reveals tissue niches and interactions in subcortical multiple sclerosis lesions	Celia Lerma‐Martin	2024	Spatial transcriptomics (**Visium**) integrated with snRNA‐seq IHC, IF, RNA‐FISH validations	Human	MS	Chronically active vs. inactive brain lesions	Immune and glial cell types mapped in a discrete lesion niche with a novel astrocyte subtype in the lesion core Transcriptomic patterns of astrocyte to glia signaling at the lesion rim and CCC between glial, immune, and vascular cells
Spatio‐temporal transcriptomics of chromothriptic SHH‐medulloblastoma identifies multiple genetic clones that resist treatment and drive relapse	Ilia Kats	2024	Spatial transcriptomics (**Visium**), FISH validations	Human & xenograft in mouse (6–10 weeks)	Treatment‐resistant SHH medulloblastoma with or without chromothripsis	Tumor tissue (neurons)	Distinct genetic clones with treatment differences inferred from SNVs and spatially mapped Chromothriptic tumors showed higher stem/proliferative signals but lower immune infiltration & differentiation Invasive tumor microtubes chromotryptic enriched
Broad rim lesions are a new pathological and imaging biomarker for rapid disease progression in multiple sclerosis	Laura Airas	2025	Spatial transcriptomics (**GeoMx**), PET imaging validations	Human	MS	Brain lesions PET‐MRI imaging group	Expressional gradient at the lesion rim with activated myeloid cells Myeloid inflammatory cell state that correlates with more rapid MS progression, visible via TSPO PET
Programs, origins, and immunomodulatory functions of myeloid cells in glioma	Tyler E. Miller	2025	Spatial transcriptomics (**Visium**) integrated with scRNA‐seq, snATAC dataset	Human	Glioma	Tumor tissue (neuroglia)	Spatially patterned myeloid immunomodulatory patterns (like inflammatory, scavenger‐immunosuppressive, etc.) Myeloid programs correlate with different spatial niches, like the scavenger immunosuppressive state enriched in hypoxic and vascular niches (which is tied to immunotherapy resistance)

*Note*: Selected recent studies from both human and mouse tissue focusing on spatial transcriptomics, proteomics, and metabolomics. Studies are ordered by year of publication for each organ.

Abbreviation: ACTC1: actin alpha cardiac muscle 1, ALS: amyotrophic lateral sclerosis, ASS: antisynthetase syndrome, CCC: cell–cell communication, CRACK: calcium imaging‐based spatial transcriptomics platform, DEGs: differentially expressed genes, DLPFC: dorsolateral prefrontal cortex, DMD: Duchenne muscular dystrophy (mdx mice), dpi: days past injury, DUX4: mouse model with facioscapulohumeral muscular dystrophy‐like pathophysiology, EAE: experimental autoimmune encephalomyelitis, ECM: extracellular matrix, FISH: fluorescence in situ hybridization, Gal‐3: galectin‐3, GeoMx: GeoMx Digital Spatial Profiler, GABAergic: gamma‐aminobutyric acid–producing (inhibitory) neurons, Gluta‐: glutamatergic (excitatory neuron subtype), HMOX1: heme oxygenase 1, H&E: hematoxylin and eosin, IBM: inclusion body myositis, IHC: immunohistochemistry, IF: immunofluorescence, IMNM: immune‐mediated necrotizing myopathy, ISH: in situ hybridization, LC‐MS: liquid chromatography–mass spectrometry, LGMD: limb‐girdle muscular dystrophy, MALDI‐MSI: matrix‐assisted laser desorption/ionization mass spectrometry imaging, MERSCOPE: multiplexed error‐robust fluorescence in situ hybridization platform, MRI: magnetic resonance imaging, MS: multiple sclerosis, NMJ: neuromuscular junction, PAX7: paired box protein Pax‐7, Penk: proenkephalin, PET: positron emission tomography, RIZs: regenerative inflammation zones, sc/snRNA‐seq: single‐cell/single‐nuclei RNA sequencing, Seq‐Scope: spatial transcriptomics platform using patterned flow cells, SHH: Sonic Hedgehog, SMPC: skeletal muscle progenitor cell, snATAC: single‐nucleus assay for transposase‐accessible chromatin, SOD1: superoxide dismutase 1 Spp1: secreted phosphoprotein 1, ST: spatial transcriptomics (adapted from Ståhl et al.), Tac: tachykinin, TOMOseq: transcriptome tomography sequencing, TSPO PET: translocator protein positron emission tomography, VSP: valosin‐containing protein (IBM like mouse model), Visium: Visium Spatial Gene Expression platform.

One of the key advantages of spatial profiling lies in its compatibility with histological analysis. For instance, Seq‐Scope spatial data (submicron resolution) were segmented with the help of H&E images to identify previously unrecognized transitional fiber states and niche specialization in denervated murine soleus muscle tissue, extending beyond classical Type 1/2 classifications [62]. Distinct myofiber types showed differential expression of heat shock proteins (HSP) and stress‐response genes following denervation. Increased macrophage and fibroblast recruitment to the neuromuscular junction (NMJ) was also observed, pointing to broader tissue‐level remodeling involving immune and stromal components. Analogous to “radiating inflammation signals” in murine injury and DMD models, denervation signals (like *Hspb1*, *Hspa8)* emanated from postsynaptic areas (marked by *Prkar1a*) of NMJs to surrounding healthy tissue.

The complexity of myofiber heterogeneity in both healthy and diseased muscle has been explored using complementary spatial modalities. In a high‐fat diet‐induced myopathy mouse model, spatial proteomics via GeoMx (NanoString) revealed aberrant expression of neuronal markers in desmin‐expressing myofibers, suggesting denervation and increased vulnerability [63]. Moreover, MSI studies have uncovered metabolic heterogeneity within and between fiber subtypes. Distinct metabolomic profiles were observed among Type 1 and Type 2B fibers in mice, including a novel “2b‐mito^high^” subtype characterized by both glycolytic and fatty acid oxidation signatures [64]. Another study integrating transcriptomics (TOMOseq) and spatial metabolomics (MSI) revealed metabolically distinct fiber zones along the central‐proximal/distal (slow‐fast fibers) axis of the murine tibialis muscle [65].

Such insights from mouse studies are increasingly being translated to human muscle biology. MSI was successfully applied to profile human muscle injury, revealing microbleeding with increased heme in degenerated regions. This contrasted increased HO‐1 in intact regions and fiber type (e.g., MyHC‐2A) specific lipid enrichment [66]. Several studies have combined scRNA‐seq and Visium to profile immune and stromal cells in diseased muscle and validate macrophage phenotypes in patient biopsies. One study identified six macrophage subpopulations, including *Spp1*‐ and *Lgals3*‐expressing subtypes in dystrophic regions of DMD mice, with human analogs detected in biopsies from patients with DMD, inclusion body myositis (IBM), and other inflammatory myopathies [26]. Similarly, another study uncovered the multilayered architecture underlying inflammation and regeneration in DMD and acute injury mouse models [67]. They defined immune‐mediated tissue remodeling by coordinated spatial zones of immune infiltration and progenitor cell activity termed “regenerative inflammation zones” (RIZs) supported through analyses of human DMD biopsies. Myeloid subtypes like growth factor expressing “GPNMB macrophages” were identified in scRNA data and mapped around damaged myofibers using integrated (deconvoluted) Visium spatial profiling.

Further supporting translational potential, xenograft models have enabled in vivo tracking of human muscle regeneration in murine hosts. In one such study, human muscle progenitor cells were engrafted into satellite cell‐depleted mice postinjury. Using GeoMx spatial transcriptomics and snRNA‐seq, the authors mapped regenerative transcriptional programs, showing how PAX7^+^ expressing progenitor cells interact with ACTC1^+^ myofibers in distinct regenerative niches [68].

Spatial omics technologies are also being directly applied to human skeletal muscle. For example, one study employed the Xenium platform with histology and IHC to spatially resolve gene expression across fiber types and tissue compartments, identifying nuclear and perinuclear transcript enrichment in Type 1 fibers [69]. However, ISS‐based profiling remains challenged by manual segmentation, particularly in polynucleated muscle fibers, due to the lack of robust automated pipelines [50]. A recent preprint addressed this issue by generating a fully annotated dataset using IMC and immunofluorescence, laying the groundwork for machine learning‐based segmentation and classification of large biopsy datasets [70].

Disease‐focused integrative studies have further revealed key spatial features of pathological remodeling in patient samples. In IBM, the combination of snRNA‐seq, Visium spatial data, and ISH validation revealed selective loss of Type 2A fibers due to genomic and proteotoxic stress [1]. The study also identified *COL15A1*‐expressing profibrotic myofibers and discrete immune niches, highlighting spatial coupling between fiber pathology and focal immune activation.

Collectively, spatial omics technologies have opened new avenues for understanding how cellular organization and intercellular communication shape muscle health and disease. These approaches have illuminated complex processes of degeneration, regeneration, and fibrosis radiating from central zones throughout healthy tissue. The spatial distribution of myogenic, FAP, and macrophage subsets would be missed by traditional methods. While translational momentum is growing [71], robust functional validation of spatially identified targets remains essential to bridge the gap between discovery and clinical application in muscular dystrophies, inflammatory myopathies, and other muscle disorders.

## 4 Spatial Mapping of Inflammation in the Central Nervous System

Over the past decade, spatial omics technologies have progressed from theoretical promise to practical utility, particularly in neuroscience. Building on the robust cell type annotation achieved through sc/snRNA‐seq, spatial transcriptomics enabled researchers to combine high‐throughput gene expression profiling with spatial resolution, allowing molecular signatures to be linked to defined anatomical structures. Initially viewed as an extension of single‐cell‐based cell type classification, the field has since evolved into a diverse and multimodal technological landscape with steadily increasing resolution and throughput. This transformation has proven especially valuable in the study of neuroinflammation, where neuron‐glia interactions are tightly regulated by spatial proximity, local microenvironmental signals, and dynamic cell states (Figure 2) [22].

Recent studies illustrate how spatial profiling can delineate the molecular, cellular, and functional architecture of the CNS with increasing granularity (Table 1). For example, large‐scale transcriptomic atlases have charted region‐specific cell types and transcriptional programs across human brain structures [24, 25, 72]. These analyses revealed that histologically defined regions also exhibit transcriptional continuity with neighboring areas. However, such studies were based on region‐specific dissections and snRNA‐seq, presuming perfect anatomical separation, a limitation addressed by smaller‐scale spatial transcriptomics studies. One such study employed Visium to investigate neocortical lamination in the human brain [23], achieving less biased gene mapping across layers and revealing Alzheimer‐associated gene enrichment in layer V. Notably, this study also facilitated transcriptomic detection beyond cell bodies, previously a challenge in CNS sequencing. In the dorsolateral prefrontal cortex, dendrites and axons were exclusively represented in a subset of Visium spots, demonstrating that spatial profiling can capture nonsomatic neural compartments. Likewise, proteomic mapping via MIBI of Alzheimer's disease (AD) human brain tissue has revealed microglial, dynamic subpopulations like CD33/CD44 high and HLA‐DR/P2RY12/ApoE low expressing cells [29]. Underscoring the value of multimodal spatial mapping to identify pathological niches within tissue of high cellular heterogeneity. Together, both large‐scale atlases and focused spatial maps provide a foundation for studying the spatiotemporal dynamics of neurodevelopment and disease.

Spatial approaches are particularly well‐suited to uncovering transient progenitor states and migratory trajectories, which are difficult to capture using bulk or dissociated methods. For instance, researchers profiled neuronal and glial lineages across the prenatal human thalamus using snRNA‐seq, then integrated MERFISH‐based ISH data to spatially resolve transcriptional states in developing regions [73]. They identified spatial gradients of glutamatergic and GABAergic neurons, some likely derived from midbrain or telencephalic precursors. Similarly, microglia showed medial‐lateral maturation gradients. Such spatial microglial cell state patterns were also observed by another group before that utilized scRNA‐seq followed by FISH and IF spatial mapping [74]. They linked cell clusters to pathological locations both in murine experimental autoimmune encephalomyelitis (EAE) and human MS samples, revealing partly evolutionary conserved transcriptomic signatures. Hence, spatial omics deciphered both molecular and cellular features that correlate with function.

Yet, functional validation remains critical for linking expression patterns to behavior, circuit activity, and pathology. A notable approach coupled two‐photon calcium imaging in mice with high‐plex FISH in the same cortical tissue, identifying Baz1a as a sensory‐driven circuit regulator that activates somatostatin‐expressing interneurons [75]. Another study in mice paired in vivo imaging with spatial transcriptomics to align gene expression with state‐dependent neuronal activity, finding that transcriptomic principal components predicted rest vs. arousal responses correlating with receptor expression and axonal localization [76]. Additional work using MERFISH spatial proximity analyses uncovered interactive murine astrocyte‐neuron niches underlying cognitive function, later validated through loss‐of‐function assays [77]. These examples show how spatial omics can bridge molecular and functional brain organization, ultimately reflecting in vivo CCC events in the context of chronic brain inflammation.

In the context of disease, spatial omics have helped map inflammation, degeneration, and immune responses onto defined brain regions. Whole transcriptome spatial sequencing identified localized molecular modules in the ventral horn of ALS mouse models, highlighting early innate immune activation and oxidative stress preceding motor neuron degeneration [28]. In the same study, human postmortem spinal cord analyses revealed a similar ventral enrichment of *GFAP*, complement, interferon, and antigen presentation genes, pointing to conserved microglial dysfunction across species. In the human ALS‐afflicted motor cortex, spatial transcriptomics revealed gene modules associated with TDP‐43 pathology and metabolic stress localized to specific cortical layers [78]. Spatially dysregulated expression of a glutamate receptor and a deubiquitinase, with increased *GRM3* in cerebellar interneurons and increased *USP47* in granule cells, pointed to localized excitotoxicity and proteostasis disturbances, although the spatial resolution of 100 µm per spot limited identification of cellular niches. Additionally, researchers looked at the metabolome using MSI in a mouse model of Parkinson's disease [79]. This translational study investigated the use of icaritin, demonstrating stabilized mitochondrial function and subsequent reduced neuroinflammation as indicated by, for example, NLRP3 inflammasome and reduced IL‐1ß release.

Glioma and glioblastoma research has also benefited from spatial approaches on human tissue. One study identified distinct myeloid programs, including a “scavenger immunosuppressive” module enriched in hypoxic/vascular niches correlating with therapy resistance [80]. Intrinsic tumor heterogeneity was profiled, showing *FAM20C*‐mediated invasive gene programs at neuron‐rich glioma borders [81]. Both studies mapped transcriptional programs using the Visium platform. Going beyond the transcriptome, MSI profiling helped identify protein signatures, correlating clinically with higher patient survival rates [82]. Standardization of COMET sequential IF (∼40 markers) now enables reproducible high‐throughput profiling across human and mouse CNS samples [83].

Together, these studies illustrate how spatially resolved transcriptomics and proteomics facilitated cell type mapping, thereby unmasking the anatomical compartmentalization of tumor heterogeneity linked to immune suppression and therapeutic resistance in brain cancers.

Inflammation in ALS is likely to be secondary to motor neuron degeneration, and in glioma, it is believed to be strongly shaped by the local disturbed microenvironment. Multiple sclerosis (MS) instead displays a heterogeneous lesion evolution, where demyelination, compartmentalized inflammation, and subsequent neurodegeneration take place. This multifocal, but also region‐specific pathology was previously profiled in human tissue through integration of snRNA‐seq to identify cellular niches with Visium, to map these cell types, and smFISH to verify inferred molecular interactions [2]. For example, this approach helped precisely profile the lesion with the presence of myelin‐phagocytosing macrophages and the identification of a ciliated astrocyte subtype that had not been reported before. CCC inference revealed interactions such as *HMGB*‐*CD163* and *CD14*‐*ITGB1*, linking astrocytes, myeloid, and endothelial cells. Subtle gene expression shifts and communication gradients in periplaque white matter paralleled earlier EAE mouse data showing parenchymal inflammatory infiltration [84]. Using ISS (CartaNA/Xenium), others tracked temporal glial activation during EAE and confirmed radial lesion organization in archival human tissue [3].

A multiplatform study integrated Visium and GeoMx with ISS to identify three transcriptional trajectories from normal‐appearing human white matter to lesion rim and core [27], proposing that rim formation may precede lesion core expansion. Another GeoMx study profiled human broad rim lesions visible via TSPO‐PET, confirming that these rims harbor distinct, myeloid‐centric inflammatory programs [85].

Future studies should focus on longitudinal spatial profiling across disease stages and therapeutic responses, integrating multimodal data, single‐cell resolution, and functional validation to fully capture MS pathogenesis and treatment effects.

## 5 Conclusion and Future Perspective

Cell identity and functional state are not fixed but exist along spatial and temporal gradients, shaped by dynamic, context‐specific interactions within the tissue microenvironment [74, 77]. Spatial profiling technologies seek to decode these gradients, ultimately aiming to construct predictive and mechanistic models of tissue physiology and pathology that integrate deep molecular information and inform clinical decision‐making [11]. While continuous technical innovation is pushing the limits of resolution, now approaching subcellular precision, this often involves trade‐offs in terms of multiplexing capacity, throughput, time, and cost [7, 12, 13, 51]. As spatial profiling matures, standardization and broader accessibility, especially through centralized platforms and core facilities, will be essential to ensure that these technologies can be widely applied across diverse tissues, disease states, and research settings [15, 52, 53].

To truly unlock the potential of spatial omics for understanding tissue function and dysfunction, and to accelerate its clinical translation, several key challenges must be addressed: (a) the current lack of standardized protocols and cross‐platform comparability [15, 50, 53, 86], (b) the need for larger cohorts and sample sizes that enable statistically meaningful insights, and (c) the integration of functional validation, such as perturbation or lineage tracing studies, to confirm biological relevance.

In skeletal muscle, spatially resolved profiling has already advanced our understanding of fiber‐type heterogeneity, regenerative dynamics, and immune–stromal–vascular crosstalk in both health and disease [1, 20, 21, 26, 62, 64, 65, 67, 68, 69, 87]. In the CNS, similar approaches have revealed spatially organized programs underlying neurodevelopment, neurodegeneration, tumor progression, and inflammation [2, 3, 23, 25, 27, 28, 29, 72, 73, 75, 77, 80, 81, 85, 88, 89, 90]. These studies not only uncover novel cell types and disease‐associated states but also begin to highlight cellular and molecular features that could stratify patients or predict disease progression.

However, the path toward clinical integration remains challenging. Current diagnostic workflows, particularly in pathology, are still largely based on classical histology and immunohistochemistry [91, 92, 93]. Therefore, candidate biomarkers emerging from spatial omics studies should ideally be detectable using established platforms, such as IHC/IF, to bridge discovery and clinical utility. A key limitation remains the small cohort sizes in most spatial studies to date, which hampers reproducibility, statistical power, and the generalization of findings [1, 2].

Looking ahead, the future of spatial omics lies in converging methodological rigor, multi‐omic integration, and clinical relevance. Building harmonized atlases of tissue pathology across large, well‐characterized patient cohorts [31], developing interoperable pipelines for analysis [19, 52], and validating key findings through targeted perturbation will be central. Importantly, as spatial technologies become more scalable and accessible, they may move from the research setting into clinical workflows [40, 83] — first as tools for biomarker validation and prognosis, and ultimately as components of precision diagnostics. The promise of spatial biology is not only to describe where things happen in tissue but to explain how and why, ultimately, paving the way for mechanism‐guided, spatially informed therapeutic strategies.

## Ethics Statement

This article did not generate any new experiments using animals or any human studies.

## Conflicts of Interest

The authors declare no conflicts of interest.

## Data Availability

Data sharing is not applicable to this article as no new datasets were generated or analyzed during the current study.
